# 

**Published:** 1850-05

**Authors:** 


No. XII. MAY. Vol. V.

### BUFFALO MEDICAL JOURNAL

AND

### MONTHLY REVIEW

OF

### MEDICAL AND SURGICAL SCIENCE.

EDITED BY AUSTIN FLINT, M. D.

BUFFALO:

PUBLISHED BY JEWETT, THOMAS & CO.

Commercial Advertiser Buildings.

1850.

